# Genome Characterization of a Novel Bisegmented Positive-Sense RNA Virus Infecting the Bean Flower Thrips (*Megalurothrips usitatus*) Reveals an Active Antiviral RNAi Response

**DOI:** 10.3390/v18090948

**Published:** 2026-08-29

**Authors:** Xitong Zhu, Heng Li, Lin Lin, Jian-Ping Chen, Yiyuan Li, Xiaodi Hu

**Affiliations:** 1 State Key Laboratory for Quality and Safety of Agro-Products, Key Laboratory of Biotechnology in Plant Protection of MARA, Zhejiang Key Laboratory of Green Plant Protection, Institute of Plant Virology, Ningbo University, Ningbo 315211, China; 2411130072@nbu.edu.cn (X.Z.);; 2 State Key Laboratory of Ecological Pest Control for Fujian and Taiwan Crops, Fujian Agriculture and Forestry University, Fuzhou 350002, China

**Keywords:** *Megalurothrips usitatus*, virus discovery, bisegmented RNA virus, virus-derived small interfering RNAs

## Abstract

By combining next-generation sequencing (NGS), reverse transcription polymerase chain reaction (RT-PCR), and rapid amplification of cDNA ends (RACE) PCR, we sequenced from adult bean flower thrips (*Megalurothrips usitatus*) the near-complete genome of a previously undescribed virus (related to Anopheline-associated C virus), which we named “Megalurothrips usitatus associated virus 1” (MUaV1). The viral genome comprises two linear, single-stranded, positive-sense RNA segments, designated RNA1 (3658 nt) and RNA2 (2041 nt), which shared 35.75% and 23.74% nucleotide identity with Anopheline-associated C virus (YP_009011225.1) and chronic bee paralysis virus (ASM62179.1), respectively. According to phylogenetic analysis, MUaV1 shares the closest evolutionary relationship with Anopheline-associated C virus, followed by chronic bee paralysis virus. A clear predominance of 22 nt vsiRNAs, characteristic of Dicer-mediated siRNA processing in insects, was observed, demonstrating that MUaV1 is capable of infecting the bean flower thrips and activating its antiviral RNAi response. Our study provides the first characterization of this virus in the bean flower thrips, enhancing our understanding of the bean flower thrips virome.

## 1. Introduction

The bean flower thrips (*Megalurothrips usitatus*, Thysanoptera: Thripidae) is a major pest of leguminous crops (Fabales: Fabaceae) in southern China, characterized by rapid reproduction, high insecticide resistance, and a small, cryptic body adapted to piercing-sucking feeding [1,2]. The microbiome of this species was recently characterized, revealing that bacteria belonging to the genera *Pantoea* and *Serratia* are the dominant members [3]. Although the bean flower thrip is known to transmit the tobacco streak virus (TSV) by carrying infected pollen and inoculating it into new hosts during feeding [4,5,6], no study has yet employed high-throughput sequencing to discover novel viruses in this species. In our study, transcriptome and siRNA sequencing were performed on adult bean flower thrips, leading to the identification of a novel single-stranded positive-sense RNA virus. The full-length genomic sequence of this virus was subsequently determined using PCR and RACE approaches.

## 2. Materials and Method

### 2.1. Insect Sampling and RNA Sequencing

A total of 100 adult bean flower thrips (*Megalurothrips usitatus*) individuals were collected at Ningbo University, Zhejiang Province, China (approximately 29.91° N, 121.63° E) in March 2025, and subsequently stored at −80 °C. The samples were then sent to Novogene Corporation (Tianjin, China) for RNA extraction and sequencing. RNA extraction was performed using the CTAB method, followed by ribosomal RNA (rRNA) removal using the NEBNext rRNA Depletion Kit (NEB #E6310, New England Biolabs, Ipswich, MA, USA) during library construction prior to sequencing. After library preparation using the NEBNext Ultra Directional RNA Library Prep Kit for Illumina (NEB #E7420, New England Biolabs, Ipswich, MA, USA) with an insert size of 250 bp, the library was sequenced on the Illumina NovaSeq 6000 platform (Illumina, San Diego, CA, USA) with 150 bp paired-end reads. A total of approximately 9.92 Gb of raw data was obtained, and after quality filtering and adapter removal using Trimmomatic (v0.39) [7], approximately 9.30 Gb of clean data remained for subsequent analysis.

### 2.2. Species Identification

Species confirmation was performed by assembling the cytochrome c oxidase subunit I (COX1) gene from short-read sequencing data using MitoZ (v3.6) [8] and searching the assembled sequence against the Barcode of Life Database (BOLD v5, https://boldsystems.org/) [9], which revealed 100% identity to the *Megalurothrips usitatus* COX1 gene (MZ882425), confirming the species as *Megalurothrips usitatus*.

### 2.3. Identification of a Novel Virus

Assembly of clean reads from the bean flower thrips using Trinity (v 2.15.1) [10] with default parameters generated 127,049 contigs, which were subsequently searched against the NCBI NR database (last accessed on 17 April 2026) via the BLASTx (v 2.14.0) algorithm implemented in DIAMOND (v2.1.6) [11].

### 2.4. Genome Structure of MUaV1

We used ORF Finder (https://www.ncbi.nlm.nih.gov/orffinder/, (accessed on 18 May 2026)) and CD-Search (https://www.ncbi.nlm.nih.gov/Structure/cdd/wrpsb.cgi, (accessed on 18 May 2026)) for open reading frame (ORF) prediction and conserved-domain annotation, respectively.

### 2.5. Phylogenetic Tree Construction

Amino acid sequences of the viral proteins and related viruses were aligned using MAFFT (v7.505) [12], filtered with trimAl (v1.5) [13], and then used to construct a maximum-likelihood phylogenetic tree using IQ-TREE (v2.3.3) [14] with 1000 bootstrap replicates.

### 2.6. VsiRNA Analysis

The same samples used for RNA-Seq library construction were also employed for sRNA sequencing. A small RNA library of bean flower thrips was constructed and subjected to single-end (50 bp) sRNA sequencing on an Illumina HiSeq 6000 platform. The raw siRNA data amounted to 889,390,100 bp. After quality control using cutadapt (v5.2) [15] with the adapter sequence “AGATCGGAAGAGCACACGTCT”, 465,140,767 bp of clean data was obtained, which was used for subsequent analyses. To explore whether the novel virus induced host antiviral pathways, the clean small RNA data was aligned to the assembled transcriptome using Bowtie2 (v2.4.5) [16].

### 2.7. Experimental Validation of the Viral Genome

To determine the complete genomic RNA sequence of the novel virus, viral contigs were first validated by RT-PCR using four primer pairs for RNA1 and two primer pairs for RNA2 (Supplementary Table S1). The sizes of the PCR products (Supplementary Figure S1) were consistent with the expected lengths, and Sanger sequencing confirmed the accuracy of the assembled viral genome sequences. To obtain the terminal sequences, 5′- and 3′-RACE were performed using commercial RACE kits (Sangon Biotech, Shanghai, China). The complete RNA2 genomic segment, including both the 5′ and 3′ untranslated regions, was successfully determined by RACE. For RNA1, only the 5′ terminal sequence could be obtained despite repeated attempts, whereas the 3′ terminus remained unresolved (Supplementary Figure S2). Nevertheless, by combining the overlapping RT-PCR fragments with the RACE-derived 5′ terminal sequence, a high-quality near-complete RNA1 genomic sequence was assembled.

## 3. Results

### 3.1. Discovery and Characterization of a Novel RNA Virus in Megalurothrips usitatus

**Figure 1:**
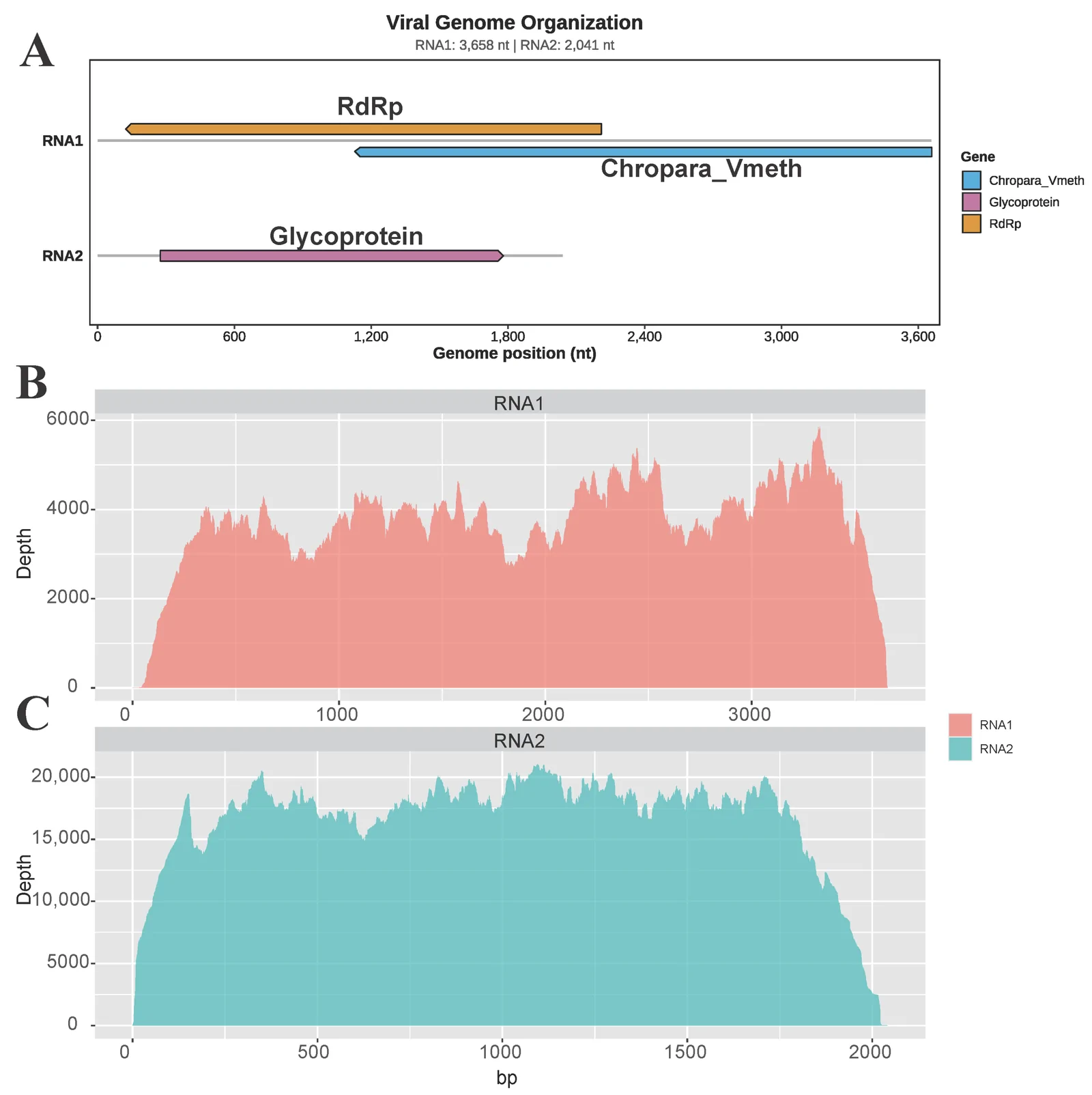
Genome architecture (**A**) and sequencing depth patterns of MUaV1 RNA1 (**B**) and RNA2 (**C**).

A total of 9.30 Gb of clean sequencing data generated 127,049 contigs. Sequence similarity searches against the NCBI non-redundant (NR) protein database identified viral-related sequences, including two contigs that potentially represented the genomic segments of a single bisegmented RNA virus (Table 1). The two contigs showed relatively low amino acid identities to their closest viral matches. The largest contig, designated RNA1, was 3658 nt in length and shared 35.75% nucleotide identity with 91% query coverage to Anopheline-associated C virus (YP_009011225.1). The second contig, designated RNA2, was 2041 nt long and exhibited 23.74% nucleotide identity with 44% query coverage to chronic bee paralysis virus (ASM62179.1). Based on these sequence similarities, the two contigs were considered to represent the genomic segments of a previously uncharacterized bisegmented RNA virus, which was designated MUaV1. As the viral sequences were derived from transcriptome sequencing and de novo assembly, the orientation of the assembled contigs does not necessarily reflect the original genomic polarity. Three putative ORFs were identified across the two genomic RNA segments: an RNA-dependent RNA polymerase (RdRp) gene on the reverse strand of RNA1 (nt 123–2210), a chromoprotein-like gene designated Chropara_Vmeth on the reverse strand of RNA1 (nt 1126–3657), and a glycoprotein gene on the forward strand of RNA2 (nt 275–1780) (Figure 1A). When mapping the clean reads back to the MUaV1 genome, we observed relatively high sequencing depth at each site, and the genome was almost fully covered by the sequencing reads (Figure 1B,C). Both RNA1 and RNA2 exhibited consistently high depth across the entire genome, with mean depths of 3698.81 for RNA1 and 16,475.60 for RNA2.

### 3.2. Phylogenetic Analysis

To further investigate the evolutionary relationships of MUaV1, phylogenetic analyses were performed based on the amino acid sequences of the RNA1-encoded RNA-dependent RNA polymerase (RdRp) and the RNA2-encoded protein. BLASTp (v 2.14.0) analysis identified the top 10 matches for the predicted RdRp and RNA2-encoded proteins, mainly to insect-associated viruses (Table 2).

**Figure 2:**
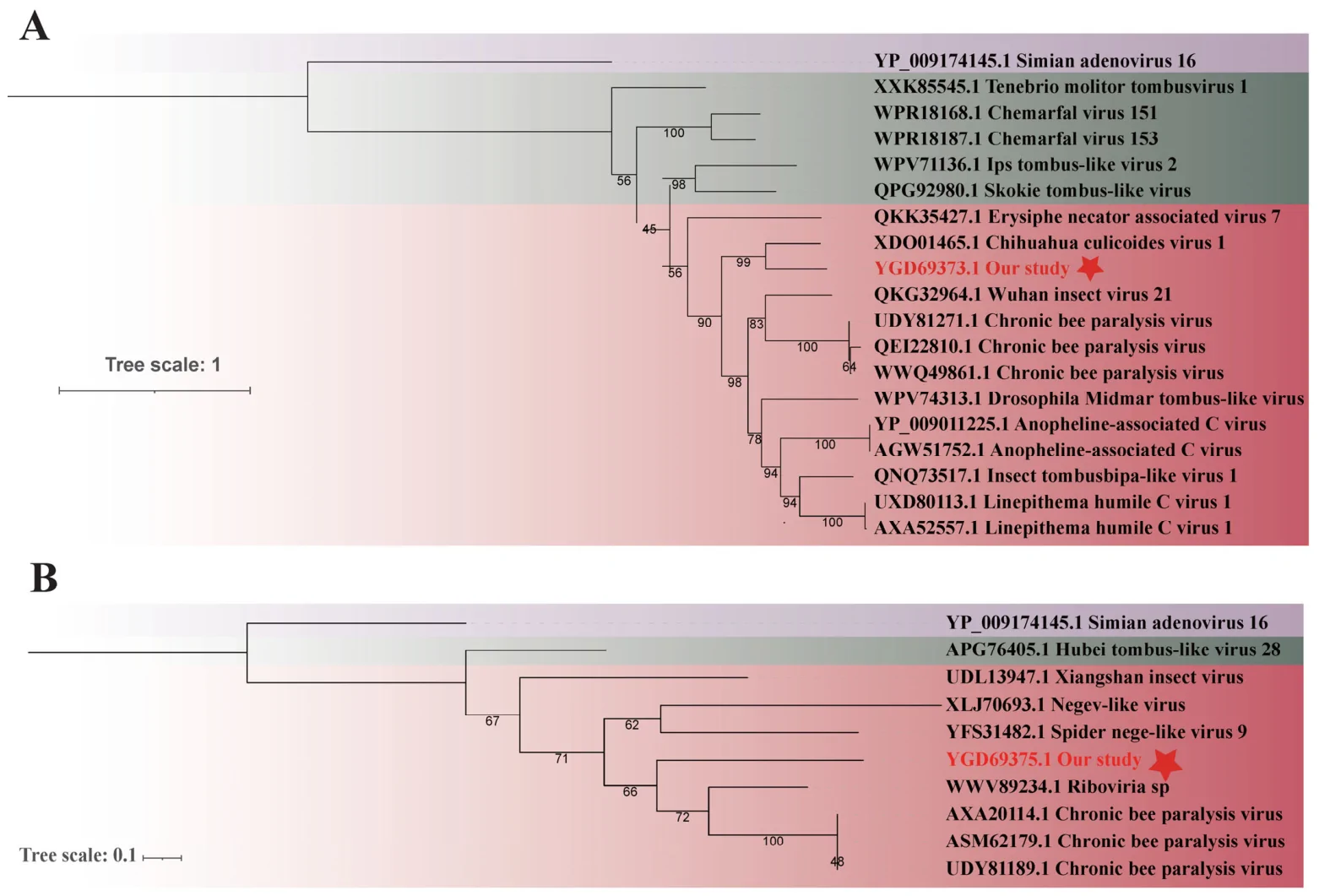
Phylogenetic analysis of MUaV1 based on the amino acid sequences of RNA1. (**A**) For the RNA1 phylogenetic analysis, the RdRp protein was compared with the following reference protein sequences: RNA1 (YGD69373.1), Simian adenovirus 16 (YP_009174145.1), Anopheline-associated C virus (AGW51752.1 and YP_009011225.1), Chronic bee paralysis virus (UDY81271.1, WWQ49861.1, and QEI22810.1), Insect tombusbipa-like virus 1 (QNQ73517.1), Linepithema humile C virus 1 (AXA52557.1 and UXD80113.1), Wuhan insect virus 21 (QKG32964.1), Drosophila Midmar tombus-like virus (WPV74313.1), Chihuahua culicoides virus 1 (XDO01465.1), Erysiphe necator associated virus 7 (QKK35427.1), Chemarfal virus 153 (WPR18187.1), Chemarfal virus 151 (WPR18168.1), Skokie tombus-like virus (QPG92980.1), Ips tombus-like virus 2 (WPV71136.1), and Tenebrio molitor tombusvirus 1 (XXK85545.1). (**B**) The RNA2 phylogenetic tree was constructed based on the predicted RNA2-encoded protein and the corresponding protein sequences from the following viruses: RNA2 (YGD69375.1), Simian adenovirus 16 (YP_009174145.1), Chronic bee paralysis virus (ASM62179.1, UDY81189.1, and AXA20114.1), Riboviria sp. (WWV89234.1), Spider nege-like virus 9 (YFS31482.1), Hubei tombus-like virus 28 (APG76405.1), Xiangshan insect virus (UDL13947.1), and Negev-like virus (XLJ70693.1). The phylogenetic tree was constructed using the maximum-likelihood method with 1000 bootstrap replicates. Bootstrap support values are shown at the corresponding nodes. The scale bar represents the number of amino acid substitutions per site. The asterisks indicate the sequences generated in this study.

In the RdRp phylogeny, MUaV1 RNA1 clustered with Chihuahua culicoides virus 1, and other insect-associated viruses, indicating a close phylogenetic relationship with these viruses (Figure 2A). The three RdRp sequences of chronic bee paralysis virus formed a well-supported cluster, consistent with their common viral origin and supporting the overall consistency of the phylogenetic reconstruction.

Phylogenetic analysis based on the RNA2-encoded protein further placed MUaV1 within a group of related insect-associated viruses (Figure 2B). Although no obvious capsid protein (CP) domain was identified in RNA2 by CD-Search, some viruses within the same phylogenetic group also lack an identifiable CP domain. Together, the phylogenetic analyses based on the two genomic segments provide evidence supporting the evolutionary affiliation of MUaV1 with related insect-associated RNA viruses [17].

### 3.3. siRNA Analysis, PCR and RACE-Based Validation of MUaV1

**Figure 3:**
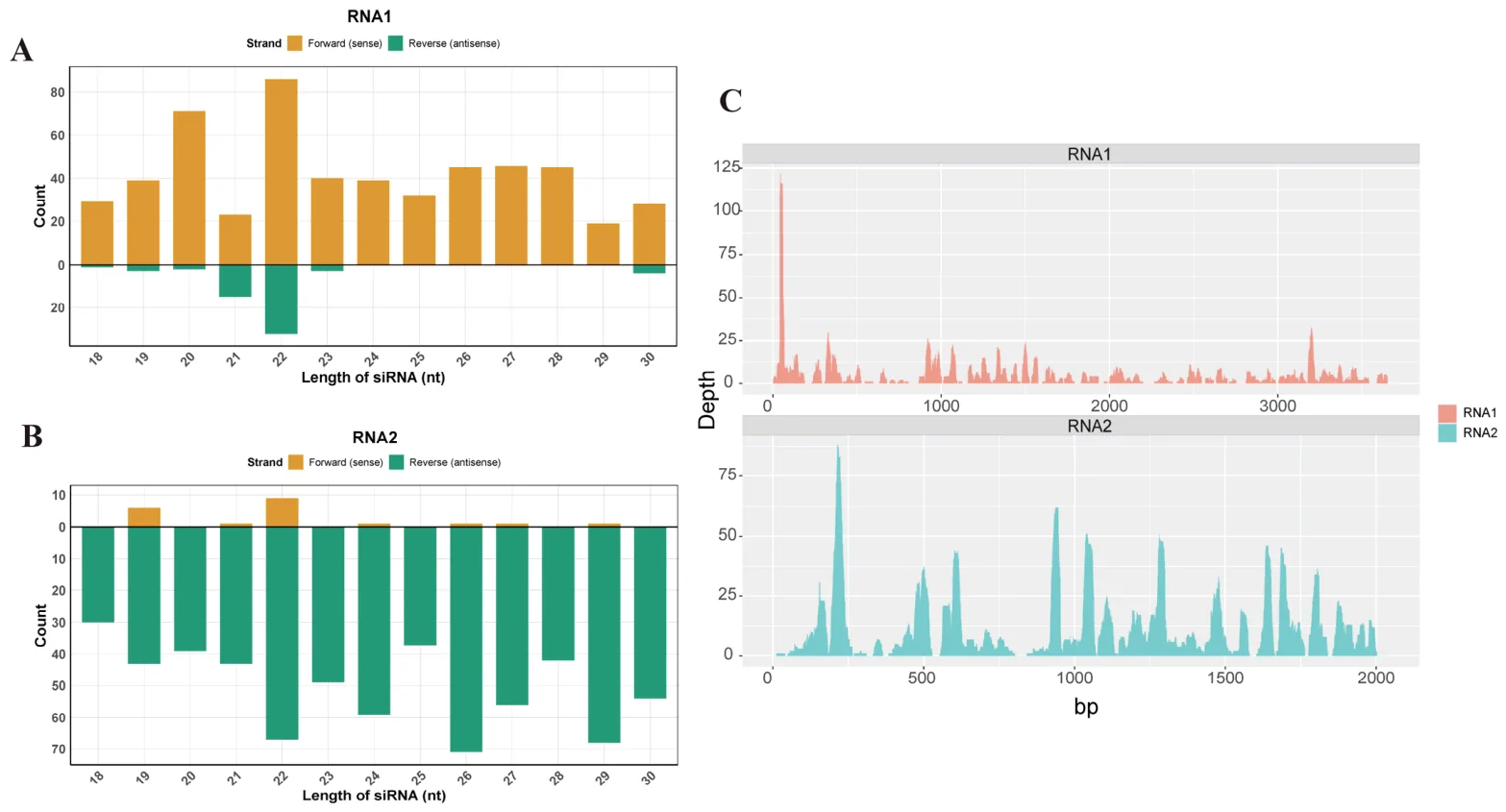
Small RNA profiles and mapping depth of MUaV1. Length distribution of small interfering RNAs (siRNAs) mapped to RNA1 (**A**) and RNA2 (**B**). (**C**) Distribution of small RNA mapping depth along the RNA1 and RNA2 genomic segments.

To better understand RNA interference (RNAi)-based antiviral immunity in response to MUaV1 infection in the bean flower thrips, we characterized virus-derived small interfering RNAs (vsiRNAs) via small RNA (sRNA) sequencing. When examining vsiRNAs aligned with the viral genome, insect-derived samples showed a prevalence of 22 nt vsiRNAs. Small RNA analysis of MUaV1 identified abundant vsiRNAs mapping to both RNA1 and RNA2. For both segments, vsiRNAs ranged from 18 to 30 nt, with 22 nt vsiRNAs being particularly abundant. RNA1 showed a predominance of forward-strand vsiRNAs (Figure 3A), whereas RNA2 was dominated by reverse-strand vsiRNAs, with major peaks at 22, 26, and 29 nt (Figure 3B). The predominant 22 nucleotide length of vsiRNAs derived from the viral genome, a typical signature of Dicer-processed siRNAs in insects, implies that the novel virus can infect insect tissue and activate the RNAi pathway of the bean flower thrips. Mapping-depth analysis showed that vsiRNAs were distributed across both RNA1 and RNA2 (Figure 3C), with several prominent accumulation hotspots, indicating that both RNA segments were targeted by the host RNA interference machinery.

RT-PCR, Sanger sequencing, and 5′/3′-RACE were performed to validate the assembled viral sequences and determine their genomic termini. These analyses confirmed the accuracy of the assembled sequences and yielded a complete RNA2 genomic segment with defined 5′ and 3′ termini, whereas RNA1 was resolved to a high-quality near-complete genomic sequence, with only the 3′ terminus remaining unresolved (Supplementary Figure S2).

## 4. Discussion

In summary, adult bean flower thrips were collected at Ningbo University, China, and the species was confirmed as *Megalurothrips usitatus* via *COX1* gene assembly. Using transcriptome assembly and small RNA (siRNA) sequencing, a novel virus in the bean flower thrips was identified, with a total genome length of 5699 bp. Although RNA1 was assembled in the opposite orientation from its inferred coding direction, this orientation is likely related to the reverse-transcription and de novo assembly process and does not necessarily indicate negative-sense genomic polarity. Thus, MUaV1 is tentatively considered a novel bisegmented positive-sense RNA virus based on its genomic characteristics and phylogenetic relationships. The viral genome was confirmed by PCR, Sanger sequencing, and RACE. Phylogenetic analyses of both genomic segments supported the evolutionary affiliation of MUaV1 with related insect-associated RNA viruses. VsiRNA analysis revealed a predominant length of 22 nucleotides, a hallmark of Dicer-processed siRNAs in insects, indicating that MUaV1 can infect insect tissue and activate the host RNAi pathway of the bean flower thrips. The strand distribution of sRNAs was determined relative to the assembled RNA1 and RNA2 sequences shown in Figure 1; however, the orientation of the assembled RNA1 contig may not reflect its actual genomic polarity. The absence of conserved terminal sequences between RNA1 and RNA2 may result from incomplete recovery or assembly of viral termini. Nevertheless, their co-occurrence in the same sample, abundant virus-derived small RNAs, and phylogenetic relationships support their association with MUaV1.

## Figures and Tables

**Table 1:** Top viral contig matches identified via online blastx (v 2.14.0) against the NCBI NR database.

Scafflod ID	Designation	Genebank No.	Scafflod Length	Reference ID	Reference Name	Identity	Coverage	Evalue	Transcriptome Depth (x)
TRINITY_DN2110_c0_g1_i1	RNA1	PZ418112	3658	YP_009011225.1	Anopheline-associated C virus	35.75%	91%	4.00 × 10^−151^	3698.81
TRINITY_DN2127_c0_g1_i1	RNA2	PZ418113	2041	ASM62179.1	Chronic bee paralysis virus	23.74%	44%	5.00 × 10^−7^	16,475.60

**Table 2:** Top 10 BLASTp (v 2.14.0) matches for the proteins predicted from MUaV1 RNA1 (RdRp, YGD69373.1) and RNA2 (Glycoprotein, YGD69375.1).

Our Genebank No.	Reference Name	Coverage	Evalue	Identity	Reference ID
YGD69373.1	Chronic bee paralysis virus	98.00%	4.0 × 10^−135^	36.43%	UDY81271.1
	Insect tombusbipa-like virus 1	96.00%	6.0 × 10^−134^	39.05%	QNQ73517.1
	Linepithema humile C virus 1	92.00%	2.0 × 10^−130^	41.42%	AXA52557.1
	Bejuita virus	97.00%	3.0 × 10^−129^	36.98%	XWX49288.1
	Drosophila Midmar tombus-like virus	98.00%	3.0 × 10^−122^	35.93%	WPV74313.1
	Anopheline-associated C virus	98.00%	1.0 × 10^−120^	36.51%	YP_009011225.1
	Wuhan insect virus 21	73.00%	3.0 × 10^−119^	42.97%	QKG32964.1
	Chihuahua culicoides virus 1	56.00%	1.0 × 10^−109^	47.84%	XDO01465.1
	Spider tombus-like virus 96	67.00%	2.0 × 10^−102^	41.67%	YFS30683.1
	Erysiphe necator associated virus 7	71.00%	3.0 × 10^−97^	37.60%	QKK35427.1
YGD69375.1	Chronic bee paralysis virus	59.00%	3.0 × 10^−7^	23.74%	ASM62179.1
	Chronic bee paralysis virus	59.00%	4.0 × 10^−7^	23.74%	UDY81189.1
	Riboviria sp.	10.00%	9.0 × 10^−5^	44.90%	WWV89234.1
	Spider nege-like virus 9	10.00%	9.0 × 10^−5^	50.00%	YFS31482.1
	Hubei tombus-like virus 28	9.00%	1.0 × 10^−4^	53.49%	APG76405.1
	Xiangshan insect virus	14.00%	1.0 × 10^−3^	30.86%	UDL13947.1
	Chronic bee paralysis virus	9.00%	6.0 × 10^−3^	43.48%	AXA20114.1
	Negev-like virus	16.00%	1.2 × 10^−2^	26.25%	XLJ70693.1
	Anopheline-associated C virus	57.00%	2.0 × 10^−1^	25.08%	YP_009011228.1
	Nephila clavipes virus 3	10.00%	2.4 × 10^−1^	42.00%	YP_009552460.1

## Data Availability

The near-complete genome sequence (accession number: RNA1, PZ418112; RNA2, PZ418113), together with the raw transcriptome and small RNA (sRNA) sequencing data (BioProject accession number PRJNA1466728), has been submitted to the NCBI database.

## Supplementary Materials

The following supporting information can be downloaded at: https://www.mdpi.com/article/10.3390/v18090948/s1, Figure S1: Electrophoresis of amplicon of RT-PCR. Lanes 1–4 show the amplification results of RNA1 with primers P1-P4; lanes 5/6 show the amplification results of RNA2 with primers P1/P2; Figure S2: Electrophoretic analysis of 3′ and 5′ RACE products from MUaV1 amplified with gene-specific primers. Primer information is available in Table S1; Table S1. Primers used in this study.
