# Supplementary material for: Genome Characterization of a Novel Bisegmented Positive-Sense RNA Virus Infecting the Bean Flower Thrips (Megalurothrips usitatus) Reveals an Active Antiviral RNAi Response
Source: Viruses. 2026 Aug 29;18(9):948. doi: 10.3390/v18090948 (PMC13611882; doi:10.3390/v18090948)
Supplement: Supplementary file 1 [file viruses-18-00948-s001.zip › viruses-4509669-supplementary.pdf]

Supplementary Table S1. Primers used in this study.

Primers	Sequence (5' to 3')	Primer binding site	Purpose
**Primers for internal sequence amplification**			
MUAV1-RNA1-P1F	CCCTCAGGAGGGAGTTACAGGT	39	Amplification of MUAV1 viral genome
MUAV1-RNA1-P1R	GCCCTTAGGTGGTTACGAGACTAC	1133	
MUAV1-RNA1-P2F	ATTAAGTAACCCATTGCACAGTGAT	867	
MUAV1-RNA1-P2R	CCACAATCTTCCAGCCTTATTAGC	1968	
MUAV1-RNA1-P3F	CAAGACCACGATGACCTGGTCT	1729	
MUAV1-RNA1-P3R	GGTACGGATTATCATCATCCATACT	2910	
MUAV1-RNA1-P4F	CTACCTTGTCCTCCTCATCCAGAG	2664	
MUAV1-RNA1-P4R	GCCCACCAGTTGTTTCTGTATC	3644	
MUAV1-RNA2-P1F	ATCATCACTGAAGCCAATGGTTTC	19	
MUAV1-RNA2-P1R	CCGTCTATCACTCTCGGGAATG	1245	
MUAV1-RNA2-P2F	CCTGGATTAAGATCAATGGTAGTTG	969	
MUAV1-RNA2-P2R	GCTTACCAAGCCCTCATACGAG	2016	
**RACE gene-specific primer**			
MUAV1-RNA1-RT1	TTTGGTTGAGACTGCTGACCTATAC	537	5' RACE reverse transcription
MUAV1-RNA1-RT2	GGCGCTCTGTCTTATAGTTTGAT	404	
MUAV1-RNA1-R1	CCGCCTAGTGTTTGAACGCTT	278	5' RACE amplification
MUAV1-RNA1-R2	CATGCGAGCCCAGATTGATATAG	183	
MUAV1-RNA2-RT1	GTGCTTAGTCTTGGAATAAATGGCT	493	5' RACE reverse transcription
MUAV1-RNA2-RT2	ACAAGCCACACACCAAGAACAG	435	
MUAV1-RNA2-R1	CAAGGGACCAGTGGCATAGAG	266	5' RACE amplification
MUAV1-RNA2-F1	CTTTGTCGAAGGACCATCCTATC	1578	3' RACE amplification
MUAV1-RNA2-F2	CTAAGCAGCGTATGCAAGTAGTGTG	1696	

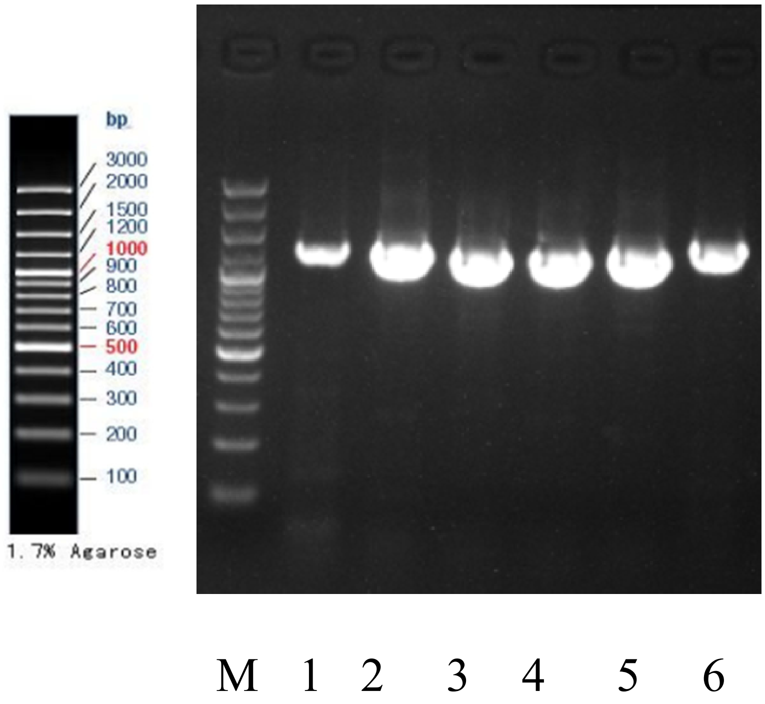

Figure S1. Electrophoresis of amplicon of RT-PCR. Lanes 1-4 show the amplification results of RNA1 with primers P1-P4; lanes 5/6 show the amplification results of RNA2 with primers P1/P2.

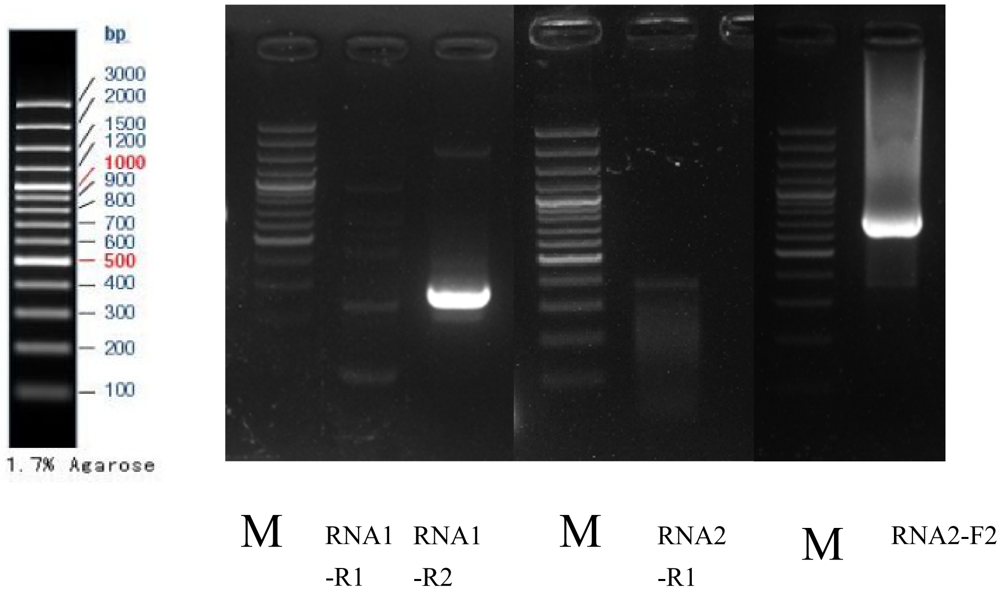

Figure S2. Electrophoretic analysis of 3' and 5' RACE products from MUaV1 amplified with gene-specific primers. Primer information is available in Table S1.
